# Prevalence of Prehypertension and Associated Cardiovascular Risk Profiles among Adults in Peru: Findings from a Nationwide Population-Based Study

**DOI:** 10.3390/ijerph19137867

**Published:** 2022-06-27

**Authors:** Akram Hernández-Vásquez, Rodrigo Vargas-Fernández

**Affiliations:** 1 Centro de Excelencia en Investigaciones Económicas y Sociales en Salud, Vicerrectorado de Investigación, Universidad San Ignacio de Loyola, Lima 15024, Peru; 2 Facultad de Ciencias de la Salud, Universidad Científica del Sur, Lima 15067, Peru; jvargasf@cientifica.edu.pe

**Keywords:** prehypertension, cardiometabolic risk factors, sex, cross-sectional studies, Peru

## Abstract

Prehypertension is a clinical condition that increases the risk of hypertension and cardiovascular disease. In South American countries, prehypertension affects almost one-third of the population. The aim of the present study was to determine the association between prehypertension and the main cardiometabolic risk factors according to the US National Cholesterol Education Program Adult Treatment Panel III by sex in the Peruvian population. A total of 863 participants surveyed were included in the study. A total of 21.1% had prehypertension, 14.4% of whom were female, and 30.5% were male. Women belonging to the age group 50–59 years, having abdominal obesity and being a current smoker, were more likely to have prehypertension, while the likelihood of having prehypertension increased in men with abdominal obesity. Three out of 10 men and one out of 10 women in Peru have prehypertension. In women, being 50 to 59 years of age, having abdominal obesity, and being a current smoker, increased the probability of having prehypertension, whereas, in men, only abdominal obesity was found to be associated with prehypertension. Our findings will allow the development of prevention strategies focused on the appropriate diagnosis of prehypertension and cardiometabolic risk factors according to sex.

## 1. Introduction

Hypertension is one of the most common chronic diseases and is recognized as a global health problem. It is one of the most important modifiable risk factors for the development of cardiovascular disease (CVD), chronic kidney disease, dementia, and a leading cause of disease burden and mortality (over eight million deaths due to hypertension) by CVD [1]. Globally, in 2019, 626 million women and 652 million men were living with hypertension, with low- and middle-income countries having the highest prevalence of this pathology [2]. One of the conditions that can progress to hypertension and is associated with cardiovascular risk is prehypertension [3]. According to the Seventh Report of the Joint National Committee on Prevention, Detection, Evaluation, and Treatment of High Blood Pressure (JNC 7), this condition is defined as systolic blood pressure (SBP) of 120 to 139 mmHg and/or diastolic blood pressure (DBP) of 80 to 89 mmHg [4]. Following diagnosis, preventive measures such as a healthy diet, weight loss, lower sodium intake, lower alcohol consumption, and physical activity have shown to be effective in delaying and preventing the development of prehypertension [5].

According to the biomedical literature, individuals with prehypertension have a two- to three-fold higher risk of developing hypertension compared to people with normal blood pressure values [6,7]. Prehypertension is also associated with an increased risk of cardiovascular disease, coronary heart disease, cerebrovascular disease, and myocardial infarction [8], as well as target organ damage such as early atherosclerosis, microvascular damage, coronary artery calcification, vascular remodeling, and left ventricular hypertrophy [9,10,11,12,13]. This increase in cardiovascular risk and the development of hypertension may vary between and within regions, with several population-based studies reporting different values of the prevalence of prehypertension based on the sociodemographic (sex and age) and economic characteristics of the populations.

Since the JNC 7 definition, epidemiological studies have reported that prehypertension is a common clinical condition affecting 25 to 50% of the general population [3], and the male sex has the highest prevalence of prehypertension [3,14,15]. In South American countries such as Argentina, Uruguay, and Chile, prehypertension affects 32.5% of the general population, with a higher prevalence in men compared to women (36.0% vs. 29.4%) [16]. In Peru, a pattern similar to that of these countries is observed, with prehypertension affecting more than 30% of the general population, and in terms of gender, the male population has a higher prevalence and probability of presenting this condition compared to women [17]. These differences in the prevalence of prehypertension according to sex may be influenced by cardiometabolic and behavioral risk factors that behave differently in men and women [18].

Several studies have reported that men and women have the same key modifiable risk factors related to prehypertension (high body mass index, high glucose levels, high levels of low-density lipoprotein [LDL], and low levels of high-density lipoprotein [HLD]) [14]. However, it has been observed that men with prehypertension have high levels of total cholesterol as one of the major risk factors for this condition, while women have high levels of triglycerides and LDL [19]. Although men in various regions of the world have a higher prevalence of prehypertension and there are differences in cardiometabolic risk factors according to sex, most epidemiological studies have focused on the prevalence of prehypertension and its traditional risk factors such as obesity and age in the general population.

In Peru, there is little information on the association between modifiable cardiometabolic risk factors and the presence of prehypertension according to gender in population-based studies. This problem increases due to cardiovascular risk and the risk of developing hypertension in persons with prehypertension, and even more so when one out of every two Peruvians is not diagnosed with hypertension despite having elevated blood pressure levels [20], and only 20.6% of hypertensive patients receive treatment for this condition [21]. Therefore, the aim of the present study was to determine the association between prehypertension and the main cardiometabolic risk factors following the US National Cholesterol Education Program Adult Treatment Panel III (NCEP-ATPIII) according to sex in the Peruvian population. 

## 2. Materials and Methods

### 2.1. Data Source, Design and Study Population

We performed a cross-sectional analytical study of data from the 2017–2018 Food and Nutrition Surveillance by Life Stages survey (VIANEV: acronym in Spanish), which was a nationally representative survey of adults aged 18 to 59 years conducted by the National Food and Nutrition Center of the National Institute of Health of Peru and its data have been used for previous studies [22,23,24,25].

The 2017–2018 VIANEV aimed to describe the nutritional status of a population of adults aged 18 to 59 years according to consumption, anthropometric and biochemical indicators in a subsample of the National Household Survey (ENAHO) of the first quarter of 2017 [22]. Likewise, the prevalence of hypertension, hyperglycemia, and hypercholesterolemia in adults is determined. The 2017–2018 VIANEV collects data from three domains: Metropolitan Lima (capital of Peru) and the remaining urban and rural areas using a stratified, multistage, probabilistic, and independent sampling process [22]. The sample selection was conducted in two stages. The primary sampling unit was a sample of randomly selected clusters, and the secondary sampling unit was a sample of randomly selected households with adults aged 18–59 years. The sample size of the 2017–2018 VIANEV was calculated at 1211 adults (men and women); at the national level, divided into 03 large strata: Metropolitan Lima (557 adults), remaining urban (256 adults), and rural areas (398 adults) [22]. In the 2017–2018 VIANEV survey, 1086 adult participants aged 18 to 59 years were visited in their households for evaluation of nutritional status and measurements. Of these, 10.3% refused to participate (16.9% in Metropolitan Lima and 3.5% and 5.3% in the remaining urban and rural areas, respectively). The National Center for Food and Nutrition of the National Institute of Health of Peru has published the detailed methodology of the 2017–2018 VIANEV, which has been described in previous studies [22,23,24].

The 2017–2018 VIANEV survey staff consisted of health science professionals, mainly nutritionists, who were trained to standardize procedures for obtaining informed consent and data collection techniques (interview), filling out and coding the questionnaire, and obtaining anthropometric measurements and biological samples [22]. Theoretical sessions, workshops, and a pilot test were conducted as part of the training.

### 2.2. Measurements and Variables

#### 2.2.1. Measurements

Determination of blood pressure, fasting blood glucose, lipid profile, waist circumference, and physical activity was performed.

In order to determine the lipid profile, serum was obtained and transported by a cold chain. For the determination of cholesterol and triglycerides, the enzymatic-colorimetric method of automated end-point coupled reactions was used. HDL and LDL were determined using the automated direct enzymatic-colorimetric method [22]. In order to determine the level of glycemia, previously calibrated portable glucometers (HemoCue Glucose 201 RT) were used. The participant was fasting (fasting should be no more than 12 h and no less than 9 h). More details on the procedures are available in the 2017–2018 VIANEV report [22].

A digital sphygmomanometer (OMRON automatic blood pressure monitor) was used to measure blood pressure [22]. The dominant arm was identified to take the blood pressure, then the blood pressure was taken twice, and an average was obtained to determine the preliminary diagnosis of blood pressure. If there was a difference of 20 mmHg between the first and second measurement of SBP and/or 10 mmHg between the first and second measurement of DBP, a new measurement was taken (third measurement), and the measurements that did not have these differences were recorded [22]. Blood pressure was measured in the morning between approximately 6:00 and 9:00 a.m., and if this was not possible, it was evaluated in the evening between 19:00 and 21:00 h [22].

Abdominal circumference was measured at the end of the exhalation using a tape measure with the person in an upright position with the torso uncovered, with the feet separated by a distance of 25 to 30 cm and at the upper edge of the iliac crest [22]. This procedure was performed three consecutive times, and the average was obtained as the final result [22].

The level of physical activity was assessed using the short version of the International Physical Activity Questionnaire (IPAQ) [22], which measures three characteristics of physical activity: frequency (days per week), duration (time per day), intensity (light, walking, moderate or vigorous) to establish three categories of physical activity: low, moderate, and high.

#### 2.2.2. Variables

The dependent variable was the presence of prehypertension, which was categorized as an SBP 120 to 139 mmHg and/or DBP 80 to 89 mm Hg coded as 1, and SBP < 120 mmHg and DBP < 80 mmHg as normal coded as 0.

The independent variables were the cardiometabolic risk factors included in the NCEP-ATPIII: age, fasting blood glucose level ≥ 100 mg/dL, HDL cholesterol < 40 mg/dL in males or <50 mg/dL in females, triglycerides ≥ 150 mg/dL, waist circumference ≥ 102 cm in males or≥88 cm in females. For the purposes of our study these factors were categorized into: age groups (18–29, 30–39, 40–49, 50–59 years), fasting blood glucose level (<100 mg/dL/ ≥ 100 mg/dL), low HDL cholesterol (yes [<40 mg/dL in males or <50 mg/dL in females]/No [≥40 mg/dL in males or ≥50 mg/dL in females]), triglycerides (<150 mg/dL/≥150 mg/dL), and abdominal obesity (Yes [when waist circumference ≥ 102 cm in males or ≥88 cm in females]/No [when waist circumference < 102 cm in males or < 88 cm in females]). Likewise, physical activity and smoking were included as independent variables as health risk factors that can have an impact on the analysis of cardiometabolic risk factors [26,27]. Physical activity was classified as low and moderate to high following the classification used in a previous study [23], and the variable current smoker (yes/no), was defined as yes when the respondent answered yes to the question “Do you currently smoke any tobacco product such as cigarettes, cigars or pipe?” and no otherwise.

The potential confounding variables identified in the scientific literature and included in our analysis were the following [17,28,29,30,31,32,33]: education level (up to primary/secondary/higher education), poverty (non-poor/poorest), health insurance status (yes/no), area of residence (urban/rural) and altitude of residence (0–499/500–1499/1500 or more). 

### 2.3. Statistical Analysis

Data were analyzed using Stata version 14 (Stata Corporation, College Station, TX, USA). The analysis considered the complex survey features: primary sampling units, strata, and sampling weights.

The characteristics of the sample participants stratified by sex were summarized using sample frequencies and complex survey features, percentages, means, and standard errors (SE).

Unadjusted prevalence ratios (PR) and 95% confidence intervals (CI) for the associations between cardiometabolic risk factors and the other study variables were obtained using bivariate Poisson regression models. The choice of this model was also motivated by the non-convergence of the log-binomial regression model. Poisson multiple regression was performed for cardiometabolic risk factors by sex with adjustments for educational level, poverty, health insurance, area of residence, and altitude of residence, and the results were considered statistically significant when the p-values were less than 0.05. Multicollinearity was assessed among the explanatory variables to be included in the Poisson regression model by variance inflation factor.

### 2.4. Ethical Considerations

Ethics committee approval was not sought as this was an analysis of secondary data that does not allow identification of the participants evaluated. The National Institute of Health provided the anonymized database of the 2017–2018 VIANEV survey data after a request for access to public information (https://web.ins.gob.pe/es/transparencia/solicitud-de-acceso-a-la-informacion-publica (accessed on 16 June 2021)). The ENAHO 2017 microdata were obtained from the INEI website (http://iinei.inei.gob.pe/microdatos/ (accessed on 18 January 2022)). In addition, as part of the 2017–2018 VIANEV, surveyors obtained informed consent from each participant for anthropometric measurements and blood sampling.

## 3. Results

A total of 863 participants surveyed were included in the study, with a predominance of females (58.2%), an age between 18 and 29 years (29.9%), having a higher educational level (42.5%), not being poor (85.7%), having health insurance (75.1%), residing in an urban area (80.4%), and at an altitude ranging from 0 to 499 plus (73.6%) (Table 1). Regarding cardiometabolic risk factors according to the NCEP-ATPIII, the mean fasting blood glucose level was 106.7 mg/dL (SE: 1.0), the mean HDL cholesterol level was 36.9 mg/dL (SE: 0.5), the average triglyceride level was 154.8 mg/dL (SE: 4.0), the mean abdominal circumference was 91.6 cm (SE: 0.5), 13.6 % were current smokers, and 61.1% had a low level of physical activity (Table 1).

Of the total number of participants, 78.9% were found to have normal blood pressure, while 21.1% had prehypertension, 14.4% were women, and 30.5% were men. In relation to the classification of normotension according to the JNC 7, significant differences were found according to age, low HDL cholesterol levels, abdominal obesity, and current smoker between men and women (Table 2). In relation to age, there was a higher proportion of women aged 30 to 59 years with normotension levels compared to men, who only surpassed women in the 18 to 29 years of age group. Likewise, a higher proportion of women were found to have low HDL cholesterol levels and abdominal obesity compared to men, while men had a higher proportion of current smokers compared to women. On the other hand, in terms of prehypertension classification, significant differences were only found in low HDL cholesterol levels and abdominal obesity, with females presenting a higher proportion of these factors compared to males (Table 2).

Considering all the participants included, the crude model showed a significant association between sex, age, triglyceride levels, and prehypertension (Table 3). Likewise, according to the sex of the participants, the crude model showed a significant association between age and triglyceride levels, abdominal obesity, and prehypertension in both sexes (Table 3). In the model adjusted for level of education, poverty, altitude of residence, and health insurance coverage, in all the participants included, being male (PR: 2.86 [95% CI: 2.04–4.00]), belonging to an age group of 50 to 59 years (PR: 2.06 [95% CI: 1.27–3.35]), having triglyceride levels ≥ 150 mg/dL (PR: 1.39 [95% CI: 1.02–1.89]) and having abdominal obesity (PR: 2.01 [95% CI: 1.48–2.73]) increased the probability of presenting prehypertension. In relation to the analysis by sex-adjusted for the same covariates, women belonging to the age group of 50–59 years (adjusted PR [aPR]: 2.90 [95% CI: 1.38–6.07]) with abdominal obesity (aPR: 2.65 [95% CI: 1.40–5.02]) and current smokers (aPR: 3. 21 [95% CI: 1.68–6.15]) had a higher probability of having prehypertension, whereas in males, having abdominal obesity (aPR: 1.74 [95% CI: 1.18–2.57]) increased the probability of having prehypertension (Table 3).

## 4. Discussion

The present study sought to determine the association between prehypertension and the main cardiometabolic risk factors following the NCEP-ATPIII according to sex in the Peruvian population. Men were found to be more frequently affected by prehypertension compared to women (30.5% vs. 14.4%). Regarding risk factors according to the NCEP-ATPIII, higher proportions of low HDL cholesterol levels and abdominal obesity were observed in females with prehypertension compared to males. Regarding cardiometabolic risk factors associated with prehypertension, women belonging to the age group of 50–59 years with abdominal obesity and current smokers were more likely to have prehypertension, whereas in men, having abdominal obesity increased the probability of having prehypertension. This association between cardiometabolic risk factors and the presence of prehypertension could be due to the method used to take blood pressure since, in our study, measurements were taken at the respondents’ homes. In a review by Htay et al. [34], they describe that blood pressure measured at home would have greater diagnostic reliability compared to ambulatory and office measurements. Likewise, it is observed that blood pressure obtained at home is one of the most adequate predictors to evaluate cardiovascular events and cardiovascular and all-cause mortality and is useful for therapeutic monitoring. In this sense, the methodology used in the 2017–2018 VIANEV survey generates greater reliability of our findings and allows us to establish the cardiometabolic profile in prehypertensive patients.

The prevalence of prehypertension in males was higher than in females. This finding is similar to that reported in studies conducted in China [15], Jamaica [35], Mongolia [36], Nepal [37], Israel [38], United States [39], Peru [17], and Taiwan [40], in which men had a higher prevalence of prehypertension compared to women. It is postulated that this difference may be attributed to biological mechanisms related to sex hormones, which have been evaluated in animal models and in humans [41]. Regarding sex hormones, it is reported that the increase in androgens in males and in animal models generates an increase in blood pressure levels [41]. In addition, female sex hormones such as estrogens are found to have cardiovascular protective effects that decrease the likelihood of increased blood pressure in premenopausal women with high estrogen levels [42]. Although there are biological factors that play an important role in the difference in the prevalence of prehypertension between men and women, other modifiable risk factors that may contribute to this difference should be considered.

Regarding risk factors according to the NCEP-ATPIII, higher proportions of low HDL cholesterol levels and abdominal obesity were observed in women with prehypertension compared to men. This difference is similar to that found in studies conducted in Russia [43], China [19], and Turkey [44], which described that women presented higher proportions of low HDL cholesterol levels and abdominal obesity compared to men. This finding may be explained by differences in weight gain experienced by women during menopause because fat storage tends to occur at the subcutaneous level, and changes in female sex hormones increase fat mass and redistribute fat to the abdomen, increasing the risk of abdominal obesity [45,46]. Likewise, in relation to low HDL cholesterol levels, in the menopausal period, women generate alterations in lipoprotein levels; however, the decrease in HDL cholesterol levels is observed in some HDL subclasses such as large HDL or in the size of HDL [47]. These cardiometabolic risk factors generate a greater predisposition of women to develop CVD and metabolic syndrome, which can increase mortality and the burden of CVD disease in women.

In women, being 50 to 59 years of age with abdominal obesity and current smokers increased the likelihood of prehypertension compared to men, in whom prehypertension is more likely only in those with abdominal obesity. These findings are similar to those reported in studies conducted in Bangladesh [48], Korea [49], and Nepal [37], which described that women over 50 years of age, with higher abdominal circumference measurements or obesity, and smokers had an increased likelihood of prehypertension. These results could be attributed to the hormonal changes that postmenopausal women undergo (low estrogen levels) [41], changes in subcutaneous fat distribution, and unhealthy lifestyles (decreased physical activity, increased fat consumption, and psychosocial factors related to stress) experienced by females compared to males [50], in whom abdominal obesity is the only risk factor for the development of prehypertension. According to the medical literature, males present a distribution of fat in visceral areas, which is strongly associated with metabolic abnormalities as opposed to the fat accumulated at the subcutaneous level observed in females [51,52]. Thus, abdominal obesity is an important risk factor for developing prehypertension in both sexes and is consistent with the results of several epidemiological studies [53,54,55,56]. Therefore, lifestyle changes and pharmacological treatments are appropriate strategies to reduce the risk of obesity in individuals and, consequently, the risk of chronic diseases such as prehypertension, hypertension, and other chronic diseases [57].

Our findings have implications for clinical practice and public health. In clinical practice, physicians in primary health care facilities should routinely measure blood pressure in individuals, especially in patients with modifiable risk factors according to the sex of the patient. This prevention strategy would help to identify patients with prehypertension and treat them in a timely manner to reduce the risk of developing hypertension or CVD over time, especially in Peru, where more than 60% of people with hypertension remain undiagnosed [20]. In public health, CVD prevention strategies should focus on early detection of prehypertension and the promotion of healthy lifestyles, assessing the risk factors of individuals according to sex in order to reduce mortality, the burden of disease due to CVD, and the high health care costs of preventable diseases.

## 5. Limitations

The present study has limitations. First, causality cannot be established between cardiometabolic factors and prehypertension because of the lack of temporality due to the cross-sectional nature of the study. Second, there could be a recall bias because the information was self-reported by the respondent and possible registry mistakes of the data by the interviewer. Furthermore, there may be a social desirability bias, as information on unhealthy lifestyles such as cigarette or alcohol consumption. Third, there are variables that have not been measured, such as menopausal status, adherence to antihypertensive medications, health status, or the presence of pre-existing comorbidities, that could serve as an adjustment to better characterize the participants included. However, the 2017–2018 VIANEV is a population-based, multistage survey that is conducted by previously trained personnel and allows characterization of the Peruvian population with prehypertension and determining their cardiometabolic risk factors.

## 6. Conclusions

Three out of 10 men and one out of 10 women have prehypertension in Peru. In women, being 50 to 59 years of age, having abdominal obesity, and being a current smoker increased the probability of having prehypertension, whereas, in men, only abdominal obesity was found to be associated with prehypertension. Our findings will allow treating physicians and public health decision-makers to implement prevention strategies focused on the adequate diagnosis of prehypertension and on the differences between modifiable risk factors according to sex in order to reduce the potential risk of developing CVD and hypertension.

## Figures and Tables

**Table 1:** Characteristics of the participants included in the study.

Characteristics	Absolute Frequency (*n* = 863)	% *
Sex		
Women	500	58.2
Men	340	41.8
Age group (years)		
18–29	247	29.9
30–39	225	25.5
40–49	208	24.1
50–59	183	20.5
Age in years, mean (SE)		37.9 (0.4)
Fasting blood glucose in mg/dL, mean (SE)		106.7 (1.0)
HDL colesterol in mg/dL, mean (SE)		36.9 (0.5)
Triglycerides in mg/dL, mean (SE)		154.8 (4.0)
Waist circunference (cm), mean (SE)		91.6 (0.5)
Smoker		
No	756	86.4
Yes	107	13.6
Level of physical activity		
Moderate/High	362	38.9
Low	501	61.1
Educational level		
Up to primary	200	17.3
Secondary	360	40.3
Higher education	303	42.5
Poverty		
Non-poor	719	85.7
Poor	144	14.3
Health insurance		
No	202	24.9
Yes	661	75.1
Area of residence		
Rural	282	19.6
Urban	580	80.4
Altitude of residence (in meters)		
0–499	602	73.6
500–1499	73	6.1
1500 or more	188	20.3

* Estimates include the weights and 2017–2018 VIANEV sample specifications; SE: standard error.

**Table 2:** Cardiometabolic risk factors according to blood pressure level, 2017–2018 VIANEV.

		Normotension				Prehypertension		
	All	Women	Men		All	Women	Men	
Characteristics	% (95% CI)	% (95% CI)	% (95% CI)	*p* Value	% (95% CI)	% (95% CI)	% (95% CI)	*p* Value
Overall	78.9 (75.8–81.7)	85.6 (81.9–88.7)	69.5 (63.9–74.7)		21.1 (18.3–24.2)	14.4 (11.3–18.1)	30.5 (25.3–36.1)	
Age group (years)								
18–29	33.6 (29.2–38.2)	29.6 (24.6–35.1)	40.4 (33.3–47.9)	0.032	16.2 (11.3–22.8)	10.9 (5.5–20.6)	19.7 (12.7–29.2)	0.095
30–39	25.8 (21.9–30.1)	26.3 (21.7–31.6)	24.8 (19.0–31.6)		24.5 (18.2–32.0)	17.6 (10.1–29.1)	28.9 (20.4–39.3)	
40–49	23.7 (20.1–27.8)	27.1 (22.3–32.4)	18.0 (13.3–24.0)		25.7 (19.3–33.5)	29.9 (19.5–42.9)	23.0 (15.4–32.8)	
50–59	16.9 (13.8–20.6)	17.0 (13.4–21.4)	16.8 (12.1–22.8)		33.6 (26.9–41.0)	41.5 (30.4–53.6)	28.4 (20.3–38.2)	
Fasting blood glucose in mg/dL								
<110	65.1 (60.3–69.6)	66.0 (60.2–71.4)	63.5 (56.0–70.4)	0.568	64.3 (56.1–71.7)	62.0 (49.7–72.9)	65.8 (55.5–74.8)	0.606
≥110	34.9 (30.4–39.7)	34.0 (28.6–39.8)	36.5 (29.6–44.0)		35.7 (28.3–43.9)	38.0 (27.1–50.3)	34.2 (25.2–44.5)	
Low HDL cholesterol								
No	22.1 (18.5–26.2)	16.4 (12.1–21.8)	31.9 (26.1–38.4)	<0.001	20.3 (14.6–27.4)	10.3 (4.7–21.0)	26.8 (18.7–36.9)	0.015
Yes	77.9 (73.8–81.5)	83.6 (78.2–87.9)	68.1 (61.6–73.9)		79.7 (72.6–85.4)	89.7 (79.0–95.3)	73.2 (63.1–81.3)	
Triglycerides in mg/dL								
<150	64.8 (60.4–68.9)	68.0 (62.3–73.2)	59.3 (51.9–66.2)	0.061	42.6 (34.9–50.6)	47.7 (35.5–60.1)	39.3 (29.7–49.7)	0.299
≥150	35.2 (31.1–39.6)	32.0 (26.8–37.7)	40.7 (33.8–48.1)		57.4 (49.4–65.1)	52.3 (39.9–64.5)	60.7 (50.3–70.3)	
Abdominal obesity								
No	62.0 (57.6–66.3)	47.0 (41.4–52.8)	87.8 (81.5–92.2)	<0.001	48.2 (39.8–56.7)	17.5 (10.2–28.5)	68.4 (57.1–77.9)	<0.001
Yes	38.0 (33.7–42.4)	53.0 (47.2–58.6)	12.2 (7.8–18.5)		51.8 (43.3–60.2)	82.5 (71.5–89.8)	31.6 (22.1–42.9)	
Smoker								
No	87.5 (84.0–90.3)	94.1 (90.8–96.3)	76.2 (69.1–82.1)	<0.001	82.2 (74.2–88.1)	87.9 (77.8–93.8)	78.4 (67.4–86.4)	0.111
Yes	12.5 (9.7–16.0)	5.9 (3.7–9.2)	23.8 (17.9–30.9)		17.8 (11.9–25.8)	12.1 (6.2–22.2)	21.6 (13.6–32.6)	
Level of physical activity								
Moderate/High	38.5 (34.1–43.2)	36.1 (30.8–41.8)	42.7 (36.1–49.6)	0.119	40.0 (32.8–47.6)	31.1 (21.4–42.7)	45.9 (35.8–56.2)	0.060
Low	61.5 (56.8–65.9)	63.9 (58.2–69.2)	57.3 (50.4–63.9)		60.0 (52.4–67.2)	68.9 (57.3–78.6)	54.1 (43.8–64.2)	

Estimates include the weights and 2017–2018 VIANEV sample specifications; CI: confidence interval.

**Table 3:** Association between cardiometabolic risk factors and prehypertension, 2017–2018 VIANEV.

Characteristics	Overall				Women				Men			
	Crude Model		Adjusted Model *		Crude Model		Adjusted Model *		Crude Model		Adjusted Model *	
	PR (95% CI)	*p* Value	PR (95% CI)	*p* Value	PR (95% CI)	*p* Value	PR (95% CI)	*p* Value	PR (95% CI)	*p* Value	PR (95% CI)	*p* Value
Sex												
Women	Ref.											
Men	2.12 (1.57–2.87)	<0.001	2.86 (2.04–4.00)	<0.001								
Age group (years)												
18–29	Ref.				Ref.				Ref.			
30–39	1.77 (1.06–2.94)	0.029	1.30 (0.78–2.17)	0.310	1.73 (0.69–4.37)	0.245	1.29 (0.50–3.32)	0.603	1.92 (1.10–3.35)	0.022	1.37 (0.76–2.48)	0.293
40–49	1.96 (1.22–3.17)	0.006	1.44 (0.90–2.29)	0.126	2.68 (1.16–6.18)	0.021	1.57 (0.70–3.55)	0.276	2.04 (1.16–3.57)	0.013	1.36 (0.77–2.41)	0.294
50–59	3.03 (1.92–4.78)	<0.001	2.06 (1.27–3.35)	0.003	4.97 (2.31–10.68)	<0.001	2.90 (1.38–6.07)	0.005	2.42 (1.39–4.21)	0.002	1.73 (0.96–3.12)	0.068
Fasting blood glucose in mg/dL												
<110	Ref.				Ref.				Ref.			
≥110	1.03 (0.76–1.38)	0.853	1.00 (0.74–1.33)	0.975	1.16 (0.73–1.86)	0.529	1.12 (0.72–1.75)	0.614	0.93 (0.64–1.35)	0.710	0.87 (0.62–1.24)	0.452
Low HDL colesterol												
No	Ref.				Ref.				Ref.			
Yes	1.09 (0.76–1.56)	0.626	1.02 (0.73–1.43)	0.897	1.60 (0.70–3.66)	0.262	1.45 (0.63–3.36)	0.385	1.19 (0.82–1.74)	0.363	0.94 (0.64–1.39)	0.754
Triglycerides in mg/dL												
<150	Ref.				Ref.				Ref.			
≥150	2.03 (1.50–2.75)	<0.001	1.39 (1.02–1.89)	0.036	2.04 (1.27–3.30)	0.003	1.25 (0.78–2.02)	0.355	1.76 (1.21–2.55)	0.003	1.44 (0.99–2.1)	0.057
Abdominal obesity												
No	Ref.				Ref.				Ref.			
Yes	1.55 (1.13–2.14)	0.007	2.01 (1.48–2.73)	<0.001	3.52 (1.93–6.41)	<0.001	2.65 (1.40–5.02)	0.003	2.09 (1.45–3.02)	<0.001	1.74 (1.18–2.57)	0.006
Smoker												
No	Ref.				Ref.				Ref.			
Yes	1.38 (0.92–2.05)	0.115	1.11 (0.73–1.69)	0.618	1.89 (0.97–3.68)	0.061	3.21 (1.68–6.15)	<0.001	0.92 (0.57–1.46)	0.711	0.91 (0.58–1.44)	0.694
Level of physical activity												
Moderate/High	Ref.				Ref.				Ref.			
Low	0.95 (0.72–1.26)	0.738	1.00 (0.75–1.31)	0.975	1.22 (0.75–1.97)	0.424	1.23 (0.74–2.05)	0.430	0.91 (0.65–1.29)	0.612	0.91 (0.64–1.29)	0.599

Estimates include the weights and 2017–2018 VIANEV sample specifications; * Adjusted for educational level, poverty, altitude of residence, health insurance, and area of residence; PR: prevalence ratio; CI: confidence interval.

## Data Availability

The National Institute of Health provided the anonymized database of the 2017–2018 VIANEV survey data, after a request for access to public information (https://web.ins.gob.pe/es/transparencia/solicitud-de-acceso-a-la-informacion-publica (accessed on 16 June 2021)). The ENAHO 2017 microdata were obtained from the INEI website (http://iinei.inei.gob.pe/microdatos/ (accessed on 18 January 2022)). The ENAHO 2017 databases can be obtained by accessing the survey query tab and selecting the “ENAHO Metodología ACTUALIZADA” survey, “Condiciones de Vida y Pobreza—ENAHO, and year “2017”.
